# Attributing Agency to Automated Systems: Reflections on Human–Robot Collaborations and Responsibility-Loci

**DOI:** 10.1007/s11948-017-9943-x

**Published:** 2017-07-18

**Authors:** Sven Nyholm

**Affiliations:** Eindhoven University of Technology, Eindhoven, The Netherlands

**Keywords:** Automation, Agency, Human–robot collaboration, Responsibility-loci, Responsibility-gaps

## Abstract

Many ethicists writing about automated systems (e.g. self-driving cars and autonomous weapons systems) attribute agency to these systems. Not only that; they seemingly attribute an autonomous or independent form of agency to these machines. This leads some ethicists to worry about responsibility-gaps and retribution-gaps in cases where automated systems harm or kill human beings. In this paper, I consider what sorts of agency it makes sense to attribute to most current forms of automated systems, in particular automated cars and military robots. I argue that whereas it indeed makes sense to attribute different forms of fairly sophisticated agency to these machines, we ought not to regard them as acting on their own, independently of any human beings. Rather, the right way to understand the agency exercised by these machines is in terms of human–robot collaborations, where the humans involved initiate, supervise, and manage the agency of their robotic collaborators. This means, I argue, that there is much less room for justified worries about responsibility-gaps and retribution-gaps than many ethicists think.

On Valentine’s Day of 2016, there was an unromantic encounter between an automated vehicle and a bus in Mountain View, California. One of Google’s “self-driving cars” crashed into the bus. Though no human being was injured, there was some damage to Google’s test-vehicle. Unlike on earlier occasions when there had been collisions between conventional cars and Google’s automated vehicles, where the blame had always been put on human drivers, this case was different. This was the first time that Google assumed responsibility for what happened. “We clearly bear some responsibility”, Google admitted in the accident-report. (Urmson 2016) Google also promised to update the software of their car to make it better at predicting the behavior of larger vehicles, like buses.

Another, more tragic incident in May of 2016 also marked a first in the history of robotic driving. A Tesla model S in “autopilot” mode collided with a truck that the Tesla’s sensors had not spotted, leading to the first fatal crash: the man riding in the Tesla vehicle was instantly killed when the truck and the car collided. Unlike Google, though, Tesla didn’t take responsibility for what happened. In a carefully worded statement, Tesla expressed sympathy for their customer’s family; but Tesla also emphasized that the “customer” is “always in control and responsible.” (Tesla 2016) Like Google, however, Tesla did promise to update their equipment. They promised to make their sensors better at spotting moving objects in strong sunlight. In a way, then, Tesla admitted that their product may have been causally responsible, at least in part, for the fatality, though they denied that they were to be assigned legal, and perhaps also moral, responsibility for what happened.

These two cases help to illustrate the general topic discussed in this paper: namely, how to allocate responsibility when automated technologies or robots harm or kill human beings. More specifically, what follows investigates the relation between the type of agency exercised in cases where robots harm/kill humans, and the question of who bears responsibility for that agency. The primary focus will be on automated cars. But automated weapons systems and responsibility for harms and deaths caused by them will also be discussed. In discussing these topics together, I follow writers such as Purves et al. (2015) and Danaher (2016), who also explore the important parallels between these different technologies and the harms they might cause.

Notably, the small but growing philosophical discussion of harms and deaths caused by robots has so far had the following features:

1. It is typically assumed, without much argument, that automated technologies exercise agency.
2. This agency is regarded as involving a high degree of autonomy/independence.
3. Much of the focus has been on locating potential responsibility-gaps, viz. cases where it is unclear or indeterminate who is morally or legally responsible for some outcome or event.
4. Much of the discussion has been about accident-scenarios and cases where the technologies don’t function in the ways they are meant to.

This paper takes an alternative approach. It looks more carefully at what kind(s) of agency we can sensibly attribute to the given types of robots. And it questions the tendency to view these types of agency as instances of autonomous or independent agency. (Cf. Bradshaw et al. 2013; Mindell 2015) Rather than focusing on potential responsibility-gaps, this paper formulates a set of questions we should be asking in allocating responsibility for the types of agency that will be discussed. And, rather than going directly to failures and accident-scenarios, this discussion takes as its starting point “normal” cases where these technologies function in the ways they are intended to.

The main thesis of this paper can be summarized as follows. Yes, it does indeed make sense to attribute significant forms of agency to many current robotic technologies, such as automated cars or automated weapons systems. But this agency is typically best seen as a type of collaborative agency, where the other key partners of these collaborations are certain humans. This means that when we try to allocate responsibility for any harms or deaths caused by these technologies, we should not focus on theories of individual agency and responsibility for individual agency. We should rather draw on philosophical analyses of collaborative agency and responsibility for such agency. In particular, we should draw on hierarchical models of collaborative agency, where some agents within the collaborations are under other agents’ supervision and authority.^1^

The discussion below begins with a brief literature review to motivate the just-made observations about the existing literature (“Brief Literature Review”). The alternative approach favored here is then introduced, and key distinctions are drawn among some important forms of more and less advanced types of agency (“Different Types of Agency”). In the next section, these kinds of agency are related to three cases: the agency of automated vehicles, small children, and normal adults. This helps to motivate thinking about the agency exercised by automated technologies in terms of human–robot collaborations, rather than in terms of individual agency (“Automated Cars Versus Small Children, and Individual Versus Collaborative Agency”). The next topic is responsibility-allocations and how to think of responsibility for the relevant kinds of collaborative agency. Rather than concluding that all types of human–robot collaborations should be treated alike when it comes to which parties are responsible, the suggestion made here is that there are certain key questions we should ask and certain key bases of responsibility we can consult when we look at specific cases. To illustrate this, the discussion briefly returns to the above-sketched Google- and Tesla-cases (“Collaborative Agency and Responsibility-Loci”). The final section summarizes the overall argument of the paper (“Conclusion”).

## Brief Literature Review

When philosophers discuss the ethics of automated systems, you will often find them making remarks such as the following:

If motor vehicles are to be truly autonomous and be able to operate responsibly on our roads, they will need to replicate… the human decision-making process. (Lin 2015, 69)

Driverless cars, like [automated weapons systems], would likely be required to make life and death decisions in the course of operation. (Purves et al. 2015, 855)^2^

Driverless systems put machines in the position of making split-second decisions that could have life or death implications. (Wallach and Allen 2009, 14)

What distinguishes [automated weapons systems] from existing weapons is that they have the capacity to choose their own targets. (Sparrow 2007, 70)

If a robot were indeed able to “replicate the human decision-making process”, “make life and death decisions”, make “split-second decisions” or “choose their own targets”, the robot would be an agent. On our ordinary conceptions of agency, making decisions and choices are key aspects of agency. Hence, these just-cited commentators all attribute agency, or decision-making capacities, to the automated systems themselves.^3^ Mark Coeckelbergh is even more explicit about this. He writes that when a human being uses an automated car, we can assume that “all agency is entirely transferred to the machine.” (Coeckelbergh 2016: 754)

Notably, it is becoming common to move from the premise that automated systems are decision-making agents to the claim that moral principles need to be programmed into these systems. (e.g. Arkin 2010; Goodall 2014; Gogoll and Müller 2016; Nyholm and Smids 2016) The idea is that if these systems are going to be making decisions, including “life and death decisions”, they should be making morally acceptable decisions. Otherwise they would pose an unacceptable danger to human beings. But this does not yet settle the question of who is responsible if and when people are harmed or killed by automated systems (Sparrow 2007).

Accordingly, much of the discussion has been about how to allocate responsibility for the decision-making that these robots are thought of as engaging in (e.g. Sparrow 2007; Hevelke and Nida-Rümelin 2015; Di Nucci and Santoni 2016; Coeckelbergh 2016). It is in these discussions that it becomes clear that not only do writers like the ones just quoted above attribute agency to the robots they are discussing. They are also attributing a highly autonomous and/or independent kind of agency to these machines. A look at some of their worries about responsibility-gaps will help to illustrate this.

Sparrow (2007), for example, discusses automated weapons systems, and argues that it is hard to identify anybody who can be held responsible for harm or deaths caused by these systems. The programmers, Sparrow argues, cannot be held responsible, because they cannot fully “control and predict” what their creations will do, for which reason it would be unfair to hold them accountable. The commanding officers, in turn, also cannot be held responsible: the actions of the automated weapons systems—or “killer robots”, as Sparrow calls them—are not fully “determined” by the orders of the commanding officers. What about the robots themselves? Sparrow thinks that they act independently, for reasons and motivations of their own. But he also thinks that because they cannot suffer from any punishments we might impose on them, it makes no sense to hold them responsible for their actions. None of the involved parties can sensibly be held responsible. The robots are acting independently, for which reason the humans cannot be held responsible. But the robots cannot respond to punishment and blame in the ways humans do, for which reason they cannot sensibly be held responsible.

Consider next Hevelke and Nida-Rümelin’s (2015) discussion of responsibility for crashes with automated vehicles. They first argue that car manufacturers shouldn’t be held responsible for crashes with automated vehicles, because this might disincentivize manufacturers from developing these cars (which would be a bad thing since there are many potential benefits to automated driving). (Cf. Marchant and Lindor 2012)^4^ This leaves us with car users or the cars themselves. Hevelke and Nida-Rümelin don’t take seriously the possibility of holding the automated systems responsible, focusing instead on users. They first argue that drivers of automated vehicles cannot be held responsible on the basis of any special duty of care. Accidents will be rare enough for it to be unfair to expect people to pay enough attention that they could reasonably be expected to step in and take over before accidents occur. What about drivers under the guise of risk-imposers? Hevelke and Nida-Rümelin think this would unfairly make people hostage to moral luck: the only difference between people whose automated cars harm others and people whose automated cars don’t is that the former suffer bad luck. (Cf. Williams 1982) Since everyone using automated cars is equal in how they create risks in using these cars—it makes sense, Hevelke and Nida-Rümelin argue, to hold all users collectively responsible under the guise of a risk-creating community. This can be done by means of a mandated insurance or tax on automated driving.

A worry one might have about this is that Hevelke and Nida-Rümelin’s solution opens up the potential for the sort of “retribution-gaps” that John Danaher (2016) discusses in a recent article on harms caused by robots. When somebody is harmed through somebody else’s agency (be it a human or a robot), people generally tend to want to find some individual or individuals who can be punished for this. But if the agency in question is a robotic agency, Danaher argues, then since robots are themselves not fit to be punished and no humans are fully responsible for their robotic agency, there is a potential “retribution gap”. In other words, people will have a strong impulse to want to punish somebody, but nobody will be an appropriate target of punishment. Many people are likely to find a general tax or mandated insurance insufficient to fill this retribution-gap.

One of the most noteworthy things about these just-sketched arguments is that robotic systems are portrayed as exercising a great deal of autonomy in the sense of not being under the control of any particular person. People, it is argued, cannot predict or control how the systems will act. And they cannot be expected to pay sufficient amounts of attention to how the systems act to be able to exercise control over them. The robots or automated systems, it is thought, will act independently, on their own. And so it would be unfair to hold any human agents responsible for the robotic agency exercised by the automated systems.

It is worth noting here that mere unpredictability and the inability to fully control a piece of technology do not by themselves appear to eliminate responsibility on the part of the user. If you (say) have a piece of equipment operating on the basis of a randomizing algorithm that you know is dangerous and that you cannot predict and control, you can very sensibly be held responsible for any harms this instrument might cause if you choose to use it. In order for it to make sense to think that there might potentially be a responsibility-gap here, it would seemingly need to be the case that the unpredictability and lack of control depend on the presence of a significant form of autonomy or agency in the technology. Therefore, in order for a robot or automated system to pose a challenge to human responsibility, it needs to be an autonomous agent in some non-trivial sense.^5^

But are most automated systems really strongly autonomous agents, who act independently, outside of human control, for which reason people cannot be held responsible for any harms the robotic systems might cause? Just what type of agency can we justifiably attribute to robotic systems, such as automated cars, and how does it relate to the human agency of the humans involved? An alternative way of thinking about these issues will now be described.

## Different Types of Agency

A 2012 report on automated weapons systems from the US military’s Defense Science Board offers a different perspective on the autonomy—or lack thereof—of robotic systems, such as automated weapons systems. In one key passage, the authors of this report write:

… there are no fully autonomous systems just as there are no fully autonomous soldiers, sailors, airmen, or Marines… Perhaps the most important message for commanders is that all machines are supervised by humans to some degree, and the best capabilities result from the coordination and collaboration of humans and machines. (US Defense Science Board 2012, 24)

Consider also the following remark, made by David Gunkel in a recent interview on his work on the ethics of robotics:

I would say that the distributed agency that we see for example with the corporation is a really good benchmark and… precedent for where things might go both in moral and legal terms with regard to our understanding of machines and their place in the world… (Gunkel 2016)

These two quotes point in a very different direction than the arguments and quotes reviewed in the previous section. They point in the direction of “distributed agency” and “coordination and collaboration of humans and machines.” How can we decide whether this is a better way to think of the agency of robotic systems like automated weapons systems or automated cars? That is, why might one think in these terms, rather than in terms of independent or individual agency? To answer this, it is a good idea to first zoom out to a slightly more abstract level and distinguish among various different more or less advanced forms of agency. We can then ask: (a) which of these forms of agency are automated systems able to exercise, and (b) are those forms of individual and independent agency, or are they rather best thought of as distinctive types of cooperative or collaborative agency?

The approach used here is a functionalist approach, on which different types of agency are primarily analyzed in terms of different functions that more or less advanced agents are able to perform. An alternative approach would be to focus instead on the idea of intentionality. We would then ask under what descriptions apparent actions can be desribed as intentional, or for what reasons the agent might be interpreted as acting. (e.g. Anscombe 1957 and Davidson 1980, chapter 1) I have no principled disagreement with that approach to the topic of agency in general. However, when it comes to the question of whether we can attribute agency to robots and automated systems, it appears better to first investigate what sorts of functions the systems can perform.^6^

Start first with the most basic type of agency there is. In an example Philip Pettit uses in a selection of his papers on group agency (e.g. Pettit 2007, 178), Pettit imagines a fairly simple robot that is able to do the following: it moves around in a room and is on the look-out for objects with a certain shape. If it finds these objects, the robot moves them around in certain ways (e.g. putting the objects into a bucket). When the robot does not encounter the relevant types of objects in the room, it keeps moving around until the relevant type of object appears. This, Pettit suggests, is an example of a simple type of agency: pursuing a goal in a way that is sensitive or responsive to the environment. However, as far as we know from this example, the robot might not be able to exercise any other type of agency, if it is put in any other context. And so the simplest possible agency is what we might call.

**Domain-specific basic agency**: pursuing goals on the basis of representations, within certain limited domains

More advanced agents—which may still be basic agents—would be able to pursue different types of goals on the basis of their representations, across different domains. But even more advanced agents would also be able to follow certain rules—certain *do*s and *don’t*s—in their pursuit of their domain-specific goals. (Pettit 1990) Their agency is constrained by rules that prevent the agents from pursuing their goals in particular ways, while permitting them to pursue these goals in other ways. Consider:

**Domain-specific principled agency**: pursuing goals on the basis of representations in a way that is regulated and constrained by certain rules or principles, within certain limited domains.

If we are playing sports, for example, we do certainly pursue certain goals relevant to whatever we’re playing (e.g. scoring a goal), but we do so in ways that respect the rules of the game. We are thereby exercising more advanced agency than if we are simply trying to put the ball or whatever it might be in a certain place.

Sticking with the sports-example, our principled agency may be undertaken under the watch of some authority (e.g. a referee) who makes sure that we stick to the rules and who might otherwise step in and stop us. In general, we may be exercising:

**Domain-specific supervised and deferential principled agency**: pursuing a goal on the basis of representations in a way that is regulated by certain rules or principles, while being supervised by some authority who can stop us or to whom control can be ceded, at least within certain limited domains.

Such agency might be called non-solipsistic or social, since it is partly defined in terms of a relation to other agents. (Cf. Dignum et al. 2014) But this is still different from what we can call:

**Domain-specific responsible agency**: pursuing goals in a way that is sensitive to representations of the environment and regulated by certain rules/principles for what to do/not to do (within certain limited domains), while having the ability to understand criticism of one’s agency, along with the ability to defend or alter one’s actions based on one’s principles or principled criticism of one’s agency.

This is different from simply being answerable to some authority. Responsible agency implies that even if others might criticize your agency and may give you reason to abort what you’re doing, this nevertheless leaves open the possibility of “standing one’s ground” on the basis of rules or principles that one thinks others could also recognize as valid bases for the regulation of action. Either way, this type of agency is responsive to other agents and their opinions. Hence responsible agency is also a socially embedded form of agency, just like supervised and deferential agency is. But an important difference is that it puts the different agents involved on a more equal footing. (Cf. Darwall 2006) The practice of discussing reasons for and against our own or others’ conduct assumes that we cannot simply order each around, but that we are instead on an equal enough level that we owe each other justifications. And it opens up for shared discussion of the principles or standards governing the agency in a way that supervised and deferential agency does not.

Obviously, these just-sketched forms of agency do not exhaust all possible more or less advanced forms of agency that it can be interesting to discuss. Kantians, for example, would probably want us to add agency involving the ability to act on self-adopted principles that we choose on the basis of thinking that the principles could be elevated to universal laws for all. (e.g. Korsgaard 2010) And some theorists would want us to also discuss ideas about under what descriptions actions are intentional, or issues relating to what reasons we can interpret agents as acting on the basis of. (Anscombe 1957; Davidson 1980, chapter 1) But the just-sketched range of different types of agency is sufficient for the purposes of this paper. It (a) helps to illustrate that there is a wide range of different types of agency whereby some forms are much more sophisticated than others, and (b) gives us enough by way of abstract theory needed to investigate whether automated systems like automated cars or automated weapons systems are agents of some more sophisticated sort, capable of acting independently. Let us now ask what types of agency such systems can exercise, taking automated cars as our main example. Having done that, we can next turn to the question of whether the relevant kind of agency is an autonomous or independent type of agency, or whether it is better regarded as a form of collaborative or coordinated agency.

## Automated Cars Versus Small Children, and Individual Versus Collaborative Agency

Does an automated car exercise domain-specific basic agency? That is, does it pursue goals in a way that is sensitive to representations of the environments, at least within certain specific domains of activity? An automated car is able to navigate its surroundings in the pursuit of traffic-goals: getting to its intended destination. It does so in a way that is sensitive to representations of its environment, namely, those generated by the car’s sensors and its internal model of the surroundings. (Urmson 2015; Nyholm and Smids 2016) So yes, it seems that we can attribute domain-specific basic agency to an automated car. (Cf. Pettit 2007, 178).

What about principled agency? An automated car is programmed to follow traffic rules strictly, and pursues its goals in a way that is restricted by the traffic rules. (van Loon and Maartens 2015) So yes, we can also attribute principled agency to an automated car—at least of a domain-specific kind, where the relevant domain is that of traffic.

Is this agency being watched over by any authority, who has to power to take over control or stop the car from doing what it’s doing, and to whom the car, therefore, is deferential? Yes. Depending on what engineering ideals are used, the details differ. (Cf. Urmson 2015; Tesla 2016) But whether it is the person in the car who is able to take over control of some or all of the aspects of the driving, or whether it is the engineers who monitor the cars’ performance and who update their soft- and hard-ware as needed—there is somebody acting as a supervisor to whom the car must always defer. And so we can conclude that the principled agency we can attribute to the car is of a supervised and deferential sort. (Cf. Mindell 2015).

What the car cannot do is to exercise responsible agency. The car cannot enter into a discussion about the reasons in favor of, or against, its actions, and it cannot take responsibility for its actions in the way that a responsible human being can.^7^ (Cf. Purves et al. 2015, 860–861) So it is not sensible and appropriate to attribute responsible agency to an automated vehicle. But we can attribute basic and principled agency of a supervised and deferential sort to the car, nevertheless.

Let us compare this case to that of a small child. The small child can pursue goals in a way that is responsive to its environment (e.g. going to the other side of the room in order to get to its toys). And it can follow rules laid down by its parents. In this way, the small child is also supervised by and deferential to its parents, who are authorities to whom the small child is answerable. But most likely, the small child is not yet a responsible agent who can articulate arguments, reasons, and principles in favor of or against its actions, and who can debate the merits of different courses of action in the way a responsible adult (e.g. the parents) are able to. Of course, the small child will soon acquire this ability, but it may not yet have it. (Cf. Bloom 2013) So in a way the agency of the small child can be compared to that of the automated car, though the agency the child can perform is much less domain-specific than that of the automated car.

Consider now another distinction, namely, that between individual agency and collaborative agency. The former refers, simply put, to doing things on one’s own, not necessarily needing the collaboration of others. The latter refers to doing things together with somebody else (some agent or set of agents). (E.g. Gilbert 1990; Pettit 2007) To relate this to our present topic of discussion, we will consider two different types of deferential and supervised agency.

To repeat, somebody exercises deferential and supervised agency if there is some authority who is watching over what the agent is doing, and is able to take over control or stop the agent from doing what he/she is doing. Such agency can either be (a) initiated by the acting agent, based on his/her own goals/wishes or (b) initiated by the other party, based on that other party’s goals or wishes.

For example, if a child is playing with its toys under its parents’ supervision, the child may be doing this because it wants to play with its toys. This is an example of deferential and supervised agency initiated by the agent. Consider by way of contrast also an example in which a parent instructs the small child to do some gardening (e.g. to rake leaves), and in which the parent is then watching over the child to make sure that it is doing the gardening in a way that conforms to how the parent wants this bit of gardening to be done. Let us now relate these two examples back to the distinction between individual and collaborative agency.

When the child plays with its toys on its own initiative, though under the supervision and authority of its parents, this is an instance of individual agency. The child is doing this on its own, though the parents have a watchful eye over the child. In contrast, when the child does some gardening on the parent’s initiative, and the parent is watching over the child to make sure that the child does the gardening in the right way, this is better thought of as an instance of collaborative agency. The child is acting in the service of a goal set by the parent, and the parent is acting as a supervisor who monitors and regulates the actions of the child (viz. the gardening the child performs). Even if the child is “doing most of the work”, this is nevertheless a collaborative agency, rather than a purely individual agency on the part of the child.

Now let us return to the sort of deferential and supervised agency that we have said that automated cars can exercise (at least in a limited domain), and let us ask whether that agency on the part of the car is self-initiated or other-initiated. We are here also interested in what this implies about whether the car is best thought of as exercising independent or individual agency, as one possibility, or whether it is better thought of as exercising dependent and collaborative agency, as another possibility.

Well, the car is not going to be choosing its own primary travel goals (e.g. going to the grocery store). The goals will instead be set by the person who wishes to travel in the car (e.g. somebody who needs to buy some groceries). Similarly, nor will the car set its goals with respect to things such as safety or traffic-rules; these goals will be set by car-designers and law-makers, etc. (Cf. Mindell 2015) So the deferential and supervised agency exercised by the car is undertaken in response to somebody else’s initiative. As such, it is exercising what is best understood as a kind of collaborative agency—even if the car might be doing “most of the work.” That is, the goals are set by another authoritative agent, and that authority is supervising the performance of the car, and would either stop the car or take over control if he/she ended up being unhappy with the way in which the car is performing its tasks. (Ibid.) Thus the car’s supervised and deferential agency is of a collaborative type. It is acting in the service of, and under the authority of, the person(s) whose goals and preferences the car is responsive to. This is not an independent or individual type of agency, even if it is a fairly sophisticated type of agency indeed. (Cf. Bradshaw et al. 2013).

Similarly, a military robot is acting on the basis of the strategic goals set by the commanding officers, and in the service of the more general overarching goals of the military operation. Its performance will be supervised by the commanding officers and also by its designers and the engineers working on it. If the military robot starts performing in ways that are deemed unsatisfactory by the commanding officers, then either the use of the robot will be discontinued or the designers and engineers will be asked to update the hardware and software of the robot. (US Defense Science Board 2012, 24) Given these parameters, we should not think of the military robot as acting in an independent way. Rather, insofar as we attribute agency to it, we should think of it as exercising supervised and deferential collaborative agency. That is, we should think of it as collaborating with the humans involved and as being under the supervision and authority of those humans.

## Collaborative Agency and Responsibility-Loci

Let us now reintroduce the question of responsibility into our discussion. To do this, let us start with another case involving an adult acting together with a child, this time not doing something as innocent as gardening, but rather something more questionable. Consider this case:

Case 1: An adult and a child are robbing a bank together, on the adult’s initiative, with the gun-wielding child doing most of the “work”. The adult is supervising the duo’s activities, and would step in and start issuing orders to the child, if this should be needed.

There are two agents involved here who are engaged in a collaboration: namely, the child and the adult. But when it comes to assigning responsibility for this collaboration in this case, it appears quite clear that there is one party who is responsible here, whereas the other may not be responsible even if the latter is doing most of the work. The adult is the party to this collaboration who we would hold responsible in this case. The adult is initiating, supervising, and managing this collaboration. And unlike the child, the adult is a fully responsible moral agent. (Cf. Richards 2010, chapter 8) So here we have a case of collaborative agency, where one party is doing most of the actual work, but where the other party to the collaboration is the collaborator who should be held responsible for this bank-robbery.

Consider now a case that is modelled on the Tesla-case mentioned in the introduction:

Case 2: A human is travelling in an automated vehicle, with the car in “autopilot” or “autonomous” mode. The human is supervising the driving, and would take over, or issue different driving-instructions, if this should be needed.

This is another case in which most of the work is not done by the responsible agent involved (viz. the human), but rather by the other party involved (in this case, the car). Given that the car’s performance is supervised by the human, and given that the human would take over, or issue different instructions, if he/she would find this necessary, it here makes sense to attribute responsibility according to the model that we saw above that Tesla favors. That is, given that the human operator here collaborates with the car in the role as a sort of an active supervisor, it makes sense to view the human party to this collaboration as being the responsible party.

Consider next a type of case that is more closely modelled on the example with a Google-car in the introduction:

Case 3: A human is travelling in an automated vehicle whose performance is monitored by the designers and makers of the car, who will update the car’s hardware and software on a regular basis so as to make the car’s performance fit with their preferences and judgments about how the car should perform in traffic.

When we think of the performance of the car in this way—i.e. as being under the close watch of the designers and makers, who will step in and update its hard- and software if they deem it necessary—it appears more intuitive to view the engineers behind the car as being key parties within the human–robot collaboration here. Viewed in this way, this appears to be a case where the people who make and then update the car are the main loci of responsibility for how the car performs when it participates within human–robot collaborations.

Let us also return to automated weapons systems. Consider the following case:

Case 4: A military robot is able to operate in “autonomous” mode. The commanding officers set the goals the robot is supposed to achieve, and will stop using the robot if its performance does not help to fulfil those goals.

Here, the robot is collaborating with the humans involved. Its goals are the military goals at issue. And the humans involved will discontinue their use of this robot if they feel that the goals are not achieved in a way they are satisfied with. As before, the robot might be doing most of the work within the collaboration, but the human is the party to whom it makes sense to attribute responsibility. (Cf. the notion of “command responsibility” in e.g. Doty and Doty 2012 and various contributions in Bhuta et al. 2015) The robot is not acting independently, but is rather collaborating with the commanding officers in a supervised and deferential way. There is a clear parallel between this case and the Tesla-type scenario in case 2 above.

Consider next:

Case 5: A military robot is able to operate in “autonomous” mode. Its designers are paying close attention to whether the commanding officers are happy with the robot’s performance. If not, the designers and engineers update the hardware and software of the robot so as make its performance better track the commanding officers’ preference and judgments about how the robot should perform.

As before, we should think of this as a human–robot collaboration—not as an independently acting robot outside of human control and responsibility. When we think of the military robot in this way as being supervised, not only by the commanding officers, but also by its designers and the engineers working on it, it makes sense to reflect more on which of the humans involved are most responsible. It makes much more sense to worry mostly about that question, rather than thinking that there might be a responsibility-gap here because an autonomous robot is acting outside of human control and oversight.

In contrast, if a military robot were to just magically materialize out of nowhere—and it suddenly entered a human battlefield and started participating in the fighting—there might be a genuine responsibility-gap where it is unclear whether any of the involved human agents are responsible. However, if a military robot is put on the battlefield by military engineers and commanding officers who are collaborating to augment the human effort with robotic agency, the key question becomes who of the human parties to these human–robot collaborations bears the most responsibility. There should be no question as to whether the humans involved in these collaborations bear a significant responsibility. Again, unless the robot appears out of thin air and starts acting in a wholly independent way within the human–robot interactions in question, it is collaborating with the humans involved. (Bradshaw et al. 2013; Mindell 2015) Consequently, there should not be any real doubt as to whether it is possible to attribute responsibility to the key human players involved.^8^

The most difficult questions here instead concern what humans are most responsible for any potential bad outcomes caused by their robot collaborators. This is especially difficult when, unlike in the military context, the humans involved are not clearly all involved in any obvious form of shared collaboration. To see this, consider the human person traveling in the car in case 3 above. And imagine that rather than what was the case in the Google-crash from the introduction, the human in the car is not part of the organization that makes and then continually updates the car.^9^ The human driver of the car may have purchased the car, but the car company still regularly monitors the performance of the cars they make, and then often update at least the car’s software, but sometimes also its hardware. This can be taken to mean the following: the car is executing the owner’s more particular travelling goals (e.g. going to the grocery store), but it does so in a way that is partly monitored by and deferential to the company who builds and updates these kinds of cars. In terms of means and ends, we might say that the traveller sets the end, but that the car company determines the means by which that end is achieved.

In such a case, there is a sense in which the car is collaborating both with its owner and with its makers. It is collaborating with the owner in the sense of helping to carry out the owner’s traveling goals. It is collaborating with the car-company in the sense of helping them to provide a service to their customer. This can make it hard to determine which of the humans involved are most responsible for the actions the car performs within the context of these collaborations. And it can make it hard to determine who is responsible for what aspects of the actions the car performs.

These are very difficult questions, and an attempt to settle them will not be made here. These questions clearly require much more extensive discussion. What I will do at this point, instead, is to suggest a few key questions that we should be discussing when we think more systematically about where the key responsibility-loci are within these kinds of human–robot collaborations. The sorts of questions we should be asking importantly include the following:

- Under whose supervision and control is a vehicle that is currently operating in “autopilot” or “autonomous” mode operating?
- Who is currently able to start, take over, or, at least, stop the car?
- Whose preferences regarding driving-style is the car conforming to while in “autopilot” or “autonomous” mode?
- Who is better situated to observe and monitor the car’s actual behavior on the road?
- Who has an understanding of the functioning of the car, at least on a “macro-level”?

We should also be mindful of who enjoys rights such as ownership-rights in relation to the car. And in addition to that, we should also be investigating the roles performed by the different humans involved with these cars. Not all human responsibilities depend on direct control or immediate agency. Many human responsibilities also depend on the enjoyment of rights (e.g. ownership-rights) and the roles we inhabit. Accordingly, considerations relating to the rights enjoyed by and the roles inhabited by the humans involved also bear on who is most responsible for the human–robot collaborations that there can be between humans and automated vehicles.^10^

Similarly, when it comes to automated military robots, we should be considering their use as human–robot collaborations and then ask the following types of questions when trying to determine who is most responsible for the actions of the robots:

- Under whose supervision and control is a military robot that is currently operating in “autopilot” or “autonomous” mode operating?
- Who is currently able to start, take over, or, at least, stop the robot?
- Whose preferences regarding functioning is the robot conforming to while in “autopilot” or “autonomous” mode?
- Who is better situated to observe and monitor the military robot’s actual behavior on the battlefield?
- Who has an understanding of the functioning of the robot, at least on a “macro-level”?

These are the kinds of questions we should be discussing more carefully so as to avoid the kind of worries about responsibility-gaps motivated by the sort of reasoning that Sparrow presents in his above-cited article.

The just-suggested sets of questions about automated cars and military robots apply to cases where certain humans have control over the automated systems, or are at least able to turn them off. And they apply to cases where it is possible to update or alter the technologies in question, according to our human judgments about what improvements might be needed. It might be asked here what we should think about cases where these conditions do not hold. That is, what if we completely lose control over the automated systems we use, or we are not able to turn them off? What if they do things that we do not want them to do, and we cannot stop them? Here are a few comments about these questions.

Firstly, when we think about responsibility for automated systems such as self-driving cars and current military robots, we should not base our accounts of responsibility for these systems that we can control on our worries about other possible scenarios featuring robots or other automated systems that we cannot control. Rather, we should differentiate between responsibility for technologies that we can control (even if only indirectly) and that we can update, on the one hand, and responsibility for possible technologies we could not control and that we could not update, on the other.

In the case of technologies such as automated cars or weapons systems, it is imperative that we design and use systems that we will be able to control and that we will be able to update or at least stop.^11^ (Mindell 2015) Those are the kinds of technologies where the analysis offered here applies, i.e. where it makes sense to think in terms of human–robot collaborations, where the humans are in charge and the robot collaborators are under human supervision. In such cases, the collaborative nature of the agency involved and the respective roles played by the humans and the robots help to determine where the loci of responsibility are to be found.

However, as noted in the introduction, we should not use a “one size fits all” approach within the ethics of robotic agency and human responsibility. We should also acknowledge that there can conceivably be cases in which humans lose control over advanced autonomous robots, where those robots cannot be seen as collaborating with humans, and where genuine responsibility-gaps do appear. The discussion above has not intended to deny this. Rather, the contention here is that in the kinds of cases we are typically confronted with when we create and use technologies such as automated cars and military robots, the proper analytic framework to use is that of collaborative agency of a hierarchical sort, where certain responsible humans occupy the roles of supervisors or commanders. We should also develop frameworks for dealing with responsibility and agency-analysis applying to cases where the technology is out of control and humans and automated systems cannot sensibly be seen as collaborating. But those are not the types of cases that have been discussed above.

## Conclusion

To conclude, we should not be too quick to attribute agency of a very independent sort to robots like automated cars or military robots. Rather, we should stop to think more carefully about (a) whether any of these robots are really exercising agency in any real sense and if so, (b) what kind of agency it makes most sense to attribute to automated robots. These questions are crucial to ask if we are to avoid the sort of worries about responsibility-gaps that we find in the literature on autonomous machines, such as Sparrow’s work on automated weapons systems and Hevelke and Nida-Rümelin’s work on automated cars. It is primarily if it would make sense to attribute a strongly independent form of autonomous agency to automated systems and robots that these kinds of worries of responsibility-gaps can plausibly be thought to arise. The mere presence of unpredictiability and a lack of direct control are not by themselves enough to create responsibility-gaps.

When it comes to self-driving cars and automated weapons systems, it has been argued above that we can indeed sensibly attribute agency to such automated machines and robots—just like many writers do. But we should not attribute an independent kind of agency to these machines. Rather, we do better to understand these robots as participating in human–machine collaborations, where the robots are acting under the supervision and authority of the humans involved. The humans involved are responsible for what the robots do for the reason that they initiate, and then supervise and manage, these human–machine collaborations. And unlike the robots involved, they are capable of morally responsible agency.

The distinctions drawn above—which have been related to robots, to small children, and to adult human beings—can be illustrated using the following table, which uses automated cars as its example of the sorts of robots we might be concerned with here:

**Table:** 

	Automated vehicle	Small child	Adult
Basic (domain-specific) agency?	Yes	Yes	Yes
Principled (domain-specific) agency:	Yes	Yes	Yes
Deferential and supervised, principled (domain-specific) agency:	Yes	Yes	Yes, but also able to perform agency that is not deferential and supervised
Responsible (domain-specific) agency:	No	No	Yes
Capable of performing individual/independent agency:	No	Yes	Yes
Capable of participating in collaborative agency:	Yes	Yes	Yes
Capable of taking on the role of a responsible authority-figure within collaborative agency:	No	No	Yes

This paper does not pretend to have settled any questions about how to properly allocate or distribute responsibility among the key humans involved in the types of human–robot collaborations discussed above. Clearly, there is much more work that needs to be done if we are to be able to come up with well-supported and comprehensive views about this. The foregoing section formulated a set of questions we should be asking and investigating further when we consider this pressing topic.

The main point that the sections above have tried to establish above is that if and when we attribute agency to robots such as automated cars or autonomous weapons systems, we should almost always think of this agency as occurring within human–robot collaborations. For the contexts discussed in this paper, that is the sort of agency that it is desirable to achieve in these particular kinds of robots. (Mindell 2015) Indeed, for these contexts, that is perhaps the only form of agency it is desirable to achieve in these robots. In other kinds of contexts, different claims may apply. For example, if you are seeking to create a robot companion—a robotic friend or lover—then it could make more sense to want to create a robot that is able to exercise a much more autonomous and independent form of agency. (Nyholm and Frank 2017) But for robots and automated systems that are meant to drive us around or to help us fight wars, what is desirable is to have machines that collaborate with us, that defer to us, and whose perfomance is supervised and managed by human beings.^12^

## Footnotes

1 As that remark suggests, a fully worked-out theory is not offered in what follows. The present aim is rather to motivate a certain approach to the analysis of responsibility for automated technologies, and to encourage further discussion of this approach.

2 It is worth noting here that later on in their article, Purves et al. explore the possibility that autonomous weapons systems are not autonomous agents in the sense seemingly required in order for responsibility-gaps to arise. (Purves et al. 2015, 867) Earlier on in their article, though, they take seriously the idea that both autonomous cars and automated weapons systems need to “make life and death decisions”, as noted in the quote above.

3 It should be granted that there can be deflationary accounts of decision-making in which decision-making comes apart from agency. However, the focus here is on non-deflationary and ordinary conceptions of agency and decision-making. On such conceptions, decision-making is a distinguishing feature of agency. I thank an anonymous reviewer for prompting me to note the potential for the two to come apart, at least on some deflationary conceptions of decision-making.

4 Hevelke and Nida-Rümelin’s first argument is problematic given that the presence of pragmatic reasons against holding manufacturers responsible do not necessarily establish that holding them responsible is intrinsically unwarranted. Indeed, a little later on in their article, Hevelke and Nida-Rümelin themselves in effect make this very point when they argue that norms relating to responsibility in liberal democracies should “not be consequentialist but deontological in nature.” (Hevelke and Nida-Rümelin 2015, 622).

5 I owe this argument to an anonymous reviewer.

6 The other approach to the analysis of agency in automated systems is interestingly pursued in (Purves et al. 2015).

7 Future robots with highly advanced AI might become able to do these things, but we are not there yet. (Cf. Nyholm and Frank 2017).

8 A reviewer posed the following challenge: if a robot kills a civilian, it might make sense to think of the commander as having deployed the robot, but it wouldn’t make sense to think of the commander as having killed the civilian. So why should the commander—or any other human—be held responsible for the killing of the civilian? One answer to this is as follows: we can justifiably understand the unit (viz. the human-robot organization) as having killed the civilian. And a commanding officer can be responsible for what the unit does. Accordingly, the observation that it does not make sense to view the commanding officer as having killed the civilian does not imply that the commanding officer cannot bear responsibility.

9 In the above-described Google-case, the two people in the car were Google-employees testing the car.

10 This is illustrated nicely in recent discussions in law reviews about how to apply existing law to the new case of automated vehicles. The legal discussion in those journals is already considering whether things such as ownership, contract-based relationships (e.g. employer/employee), and other things not directly relating to immediate agency can ground legal responsibility for harms caused by automated vehicles. There are a lot of interesting lines of arguments in those discussions that ethicists working on moral responsibility of robot-caused harms can draw on. See, for instance, Peterson 2012; Guerney 2013, 2015 and 2016; Ravid 2014.

11 The immediate user—e.g. the person riding in an automated car—may him- or herself not need to be able to exercise much control. Within certain technological solutions, it may be best if the immediate user does not question or second-guess the apparent decisions of the automated system. But there needs to be and will be other humans—e.g. the car company offering the service—who keep a close watch on and who control the operation of these machines. There also needs to be monitoring and updating of the information- and communication-networks that the automated system uses to navigate in the world. Google’s self-driving cars, for example, rely very heavily on Google’s map network, which they constantly need to update and maintain. Hence even if automated systems such as self-driving cars may appear “autonomous” to their immediate users, they most typically operate within a larger collaborative network within which many humans play key supervisory and managerial roles (Mindell 2015).

12 For their valuable feedback, I am grateful to two anonymous reviewers for this journal and audiences in Eindhoven, Enschede, Galway, Groningen, the Hague, and Utrecht.
